# Sertraline for Functional Recovery After Acute Ischemic Stroke: A Prospective Observational Study

**DOI:** 10.3389/fneur.2021.734170

**Published:** 2021-10-05

**Authors:** Isabella Stuckart, Timo Siepmann, Christian Hartmann, Lars-Peder Pallesen, Annahita Sedghi, Jessica Barlinn, Heinz Reichmann, Volker Puetz, Kristian Barlinn

**Affiliations:** Department of Neurology, University Hospital Carl Gustav Carus, Technische Universitaet Dresden, Dresden, Germany

**Keywords:** stroke, outcome, functional recovery, motor recovery, SSRI, sertraline, post-stroke depression, neuroplasticity

## Abstract

**Background:** Neuroprotective and neurorestorative effects have been postulated for selective serotonin-reuptake inhibitors (SSRI). We hypothesized that sertraline, which is characterized by less severe adverse effects and more stable pharmacokinetics than classic SSRI, is associated with improved functional recovery in acute ischemic stroke patients with motor deficits.

**Methods:** Prospective observational study of consecutive acute ischemic stroke patients who received sertraline for clinically suspected post-stroke depression (PSD) or at high risk for PSD. Eligibility comprised acute motor deficit caused by ischemic stroke (≥2 points on NIHSS motor items) and functional independence pre-stroke (mRS ≤1). Decision to initiate treatment with SSRI during hospital stay was at the discretion of the treating stroke physician. Patients not receiving sertraline served as control group. Favorable functional recovery defined as mRS ≤2 was prospectively assessed at 3 months. Multivariable logistic regression analysis was used to explore the effects of sertraline on 3-months functional recovery. Secondary outcomes were frequency of any and incident PSD (defined by BDI ≥10) at 3 months.

**Results:** During the study period (03/2017–12/2018), 114 patients were assigned to sertraline (*n* = 72, 62.6%) or control group (*n* = 42, 37.4%). At study entry, patients in sertraline group were more severely neurologically affected than patients in the control group (NIHSS: 8 [IQR, 5–11] vs. 5 [IQR, 4–7]; *p* = 0.002). Also, motor NIHSS scores were more pronounced in sertraline than in control group (4 [IQR 2–7] vs. 2 [IQR 2–4], *p* = 0.001). After adjusting for age and baseline NIHSS, multivariable regression analysis revealed a significant association between sertraline intake and favorable functional outcome at 3 months (OR 3.10, 95% CI 1.02–9.41; *p* = 0.045). There was no difference between both groups regarding the frequency of any depression at 3 months (26/53 [49.1%] vs. 14/28 [50.0%] patients, *p* = 0.643, BDI ≥10). However, fewer incident depressions were observed in sertraline group patients compared to patients in control group (0/53 [0%] vs. 5/28 [17.9%] patients, *p* = 0.004).

**Conclusions:** In this non-randomized comparison, early treatment with sertraline tended to favor functional recovery in patients with acute ischemic stroke. While exploratory in nature, this hypothesis needs further investigation in a clinical trial.

## Introduction

Stroke is the most common cause of acquired disability in adulthood (1). While acute stroke care has undergone major therapeutic achievements in recent years, only a few pharmaceutical agents eventually showed promising clinical results from a neuroprotective perspective (2–4). Both neuroprotective and neurorestorative effects are postulated for selective serotonin-reuptake inhibitors (SSRI) (5–7), which were suggested to promote functional recovery after stroke by modifying ischemia-associated hyperexcitation, post-stroke inflammation, and hippocampal neurogenesis. Moreover, SSRI may augment cerebral blood flow and counteract evolution of the infarct core (6–11). Animal studies have further suggested that hippocampal expression of neurotrophins such as brain-derived neurotrophic factor (BDNF), an important mediator of cerebral plasticity and neurogenesis, is stimulated by SSRI (7, 8, 12–14).

The randomized controlled fluoxetine for motor recovery after acute ischaemic stroke (FLAME) trial, published in 2011, examined the effect of the SSRI fluoxetine on motor outcome in patients with ischemic stroke. There was an improvement in motor deficits and overall functional outcome compared to placebo after 90 days in patients receiving fluoxetine (5). Meanwhile, three larger multicenter randomized controlled trials concluded that single daily intake of fluoxetine for a period of 6 months has no beneficial effect on functional outcome in patients with ischemic stroke (15–17). However, previous clinical research has mainly focused on the SSRI fluoxetine, despite promising results from the pre-clinical setting with other SSRI (18). In any case, the use of fluoxetine in stroke patients appears debatable. On the one hand, it has a higher potential for detrimental interactions with the cytochrome P450 isoenzyme system compared with other SSRI bearing a high risk of adverse drug effects especially in the elderly and in patients exposed to polypharmacy (19). On the other hand, fluoxetine is the only SSRI considered to be unsuitable for geriatric patients (20). Lastly, fluoxetine seems to be inferior to other SSRI in terms of effectiveness in the treatment of depression (21), an entity frequently found in stroke survivors (21, 22). Almost one-third of all stroke patients develops a post-stroke depression at any time after stroke and its presence is associated with unfavorable outcome regardless of stroke severity and degree of disability (23). Pharmacotherapeutic approaches using antidepressants continue to play an important role in prevention and therapy of PSD (22).

In view of the former considerations, we aimed to explore the effects of sertraline on functional outcome and development of PSD in patients with acute ischemic stroke. As clinical data on potential effects of sertraline on motor recovery in ischemic stroke are limited (18), the results could serve as basis for sample size estimation for a randomized controlled trial.

## Methods

### Study Design and Participants

This was a prospective, non-interventional observational study that enrolled consecutive patients with acute ischemic stroke at a tertiary care stroke center. Patients were eligible if they were ≥18 years, had an imaging-confirmed diagnosis of acute ischemic stroke with a corresponding motor deficit (defined as ≥2 points in the motor items of the National Institutes of Health Stroke Scale [NIHSS] score) and a new prescription of sertraline following the index event. Patients with no corresponding prescription served as the control group. Indications for sertraline grounded on routine clinical practice and comprised treatment of patients with clinically suspected PSD and patients at high risk of PSD (e.g., severe motor deficit, functional dependency, history of depression). The ultimate decision to prescribe sertraline was at the discretion of the treating stroke neurologist and independent of study-specific procedures (i.e., results from the depression instruments). Patients were not eligible for this observational study, if they had no or only mild motor deficit, a premorbid modified Rankin Scale (mRS) score ≥2 or co-morbidities likely associated with limited participation in follow-up tests (e.g., aphasia or severe dementia [defined as Mini Mental State Exam [MMSE] <10]).

As standard-of-care, patients were prescribed sertraline 50 mg daily following admission paralleled by physical therapy. Patients who met the above-mentioned study criteria, but for whom sertraline was not prescribed for clinical reasons, were assigned to the control group. Reasons for clinical decision against prescription of sertraline were: (1) lack of clinical indication as judged by the treating stroke neurologist; (2) denial of sertraline medication by the patient; (3) medical contraindications for sertraline. Patients in the control group received the same in-hospital stroke care standards but no antidepressant medication.

### Study Procedures

At study entry, patient-related (such as demographic information, previous comorbidities, medication) and stroke-related (such as onset of symptoms, etiology, localization, acute reperfusion therapies) data were recorded. Neurological and functional status (NIHSS, pre-morbid mRS), depressive symptoms (Beck Depression Inventory [BDI]) and neurocognitive function (MMSE) were evaluated. At discharge, NIHSS and mRS scores were obtained by assessors blinded to group assignment. A structured telephone interview was done at 3 months (90 ± 14 days) to evaluate the primary endpoint of this study (mRS). During the interview, further questions were asked about behavior of taking sertraline since discharge and, if applicable, the date and reason for its discontinuation. Any new vascular events that occurred in the meantime, such as stroke, transient ischemic attack (TIA), or myocardial infarction, were documented. Each patient was informed that a survey for re-evaluation of a depression including BDI and the Patient Health Questionnaire (PHQ-9) would be sent by mail. If the survey was not returned within 2 weeks, a reminder call was undertaken, which was repeated every 2 weeks. The surveys received were evaluated and the completion date recorded. The same number of study visits and interviews were accomplished at pre-specified time points for all patients, regardless of their group assignment.

### Outcome Measures

The primary outcome measure was favorable functional status at 3 months (90 ± 14 days) defined as an mRS score ≤2. Secondary outcome measures comprised frequency of any PSD (defined by BDI ≥10 or PHQ-9 ≥10 points) and incident PSD (defined as *de novo* increase of BDI ≥10 or PHQ-9 ≥10 points) at 3 months. Moreover, safety outcomes including recurrent ischemic or hemorrhagic stroke, recurrent TIA, myocardial infarction, and death were assessed at 3 months.

### Statistical Analysis

STATA software was used for statistical analysis (Version 12.1, StataCorp, College Station, TX). Non-parametric continuous data were identified by the Shapiro-Wilk test and presented as median (interquartile range, IQR). Categorical data were summarized using frequencies and percentages. Wilcoxon rank sum test was used for between-group comparisons for differences in age, time intervals, premorbid mRS, baseline NIHSS, BDI, and MMSE scores as well as follow-up PHQ-9 and BDI scores. Pearson's chi-square test or Fisher's exact test (when expected values were <5) were used for comparing sex, comorbidities, antidepressant premedication, acute stroke therapies, stroke etiologies, and presence of any depression at baseline and follow-up as well as safety and functional outcomes at 3 months among both groups. The McNemar's test was applied for within-group comparisons of differences in favorable functional outcome and the presence of any PSD between discharge and follow-up. Adjusted odds ratio (OR) including its 95% confidence interval (95% CI) was calculated by multivariable regression analysis to describe the effect of sertraline on favorable functional outcome and PSD at 3 months. Independent predictor variables entered into the final model were identified using the backward stepwise elimination method with removal set for variables with *p*-value >0.2. The significance level was set at α <0.05. Available case analysis was applied where data were missing.

## Results

**Figure 1:**
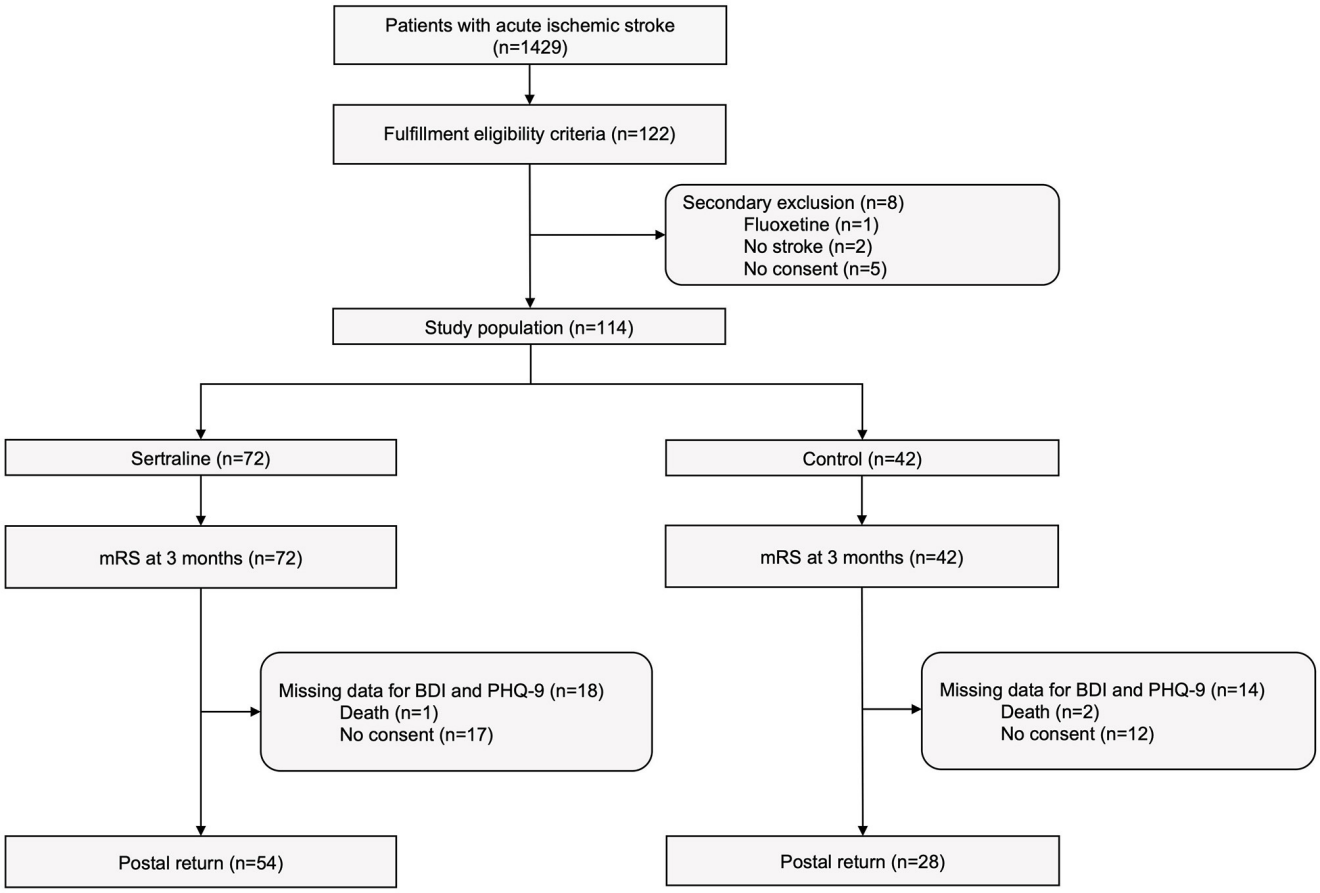
Study flow chart.

Between March 13th, 2017, and December 28th, 2018, 1429 patients with acute ischemic stroke were treated at the Department of Neurology at the University Hospital Dresden of whom 122 met study criteria and agreed to participate in this observational study. Major reasons for non-eligibility were premorbid functional dependency (mRS ≥2), lack of qualifying motor deficit, and limited ability to participate in the study (e.g., due to aphasia). Eight patients were excluded after study enrollment leaving a final study population of 114 patients, of whom 72 patients were assigned to sertraline group and 42 patients to control group. Reasons for secondary study exclusion were (1) prescription of fluoxetine (*n* = 1), (2) non-stroke diagnosis (*n* = 2), (3) withdrawal of consent to study participation (*n* = 5). The study flow chart is depicted in Figure 1.

Baseline characteristics were well-balanced between the two groups except for NIHSS that was higher in the sertraline than in the control group (8 [IQR 5–11] vs. 5 [IQR 4–7]; *p* = 0.002). Likewise, the motor items on NIHSS were more pronounced in the sertraline than in the control group (4 [IQR 2–7] vs. 2 [IQR 2–4], *p* = 0.001). The median age of patients at baseline was 70.5 (IQR 58–79) years, 48 (42.1%) patients were women, and all patients were previously functional independent. Elapsed time between symptom onset and first intake of 50 mg sertraline was 4 (IQR 3–7) days. Due to depressive symptoms, the dose of sertraline was modified during hospitalization to 75 mg (*n* = 1) or 100 mg (*n* = 5). No difference regarding the presence of depressive symptoms at study inclusion (BDI ≥10 points) was evident between both groups (32/72 [44.4%] vs. 15/42 [35.7%]; *p* = 0.936). Baseline characteristics are shown in Table 1.

**Table 1:** Baseline characteristics of the study cohort.

	**Sertraline**	**Control**	***p***
	**(*n* = 72)**	**(*n* = 42)**	
**Demographics**			
Age, years, median (IQR)	69.5 (19.5)	71 (21)	0.95
Female, *n* (%)	29 (40.3)	19 (45.2)	0.61
Premorbid mRS, *n* (%)			
0	64 (88.9)	40 (95.2)	
1	8 (11.1)	2 (4.8)	0.32
**Comorbidities/patient history**			
Risk factors, *n* (%)			
Arterial hypertension	57 (79.2)	31 (73.8)	0.51
Hyperlipidemia	40 (55.6)	18 (42.9)	0.19
Arterial fibrillation	22 (30.6)	8 (19.1)	0.20
Depression	4 (5.6)	3 (7.1)	0.71
Diabetes mellitus	21 (29.2)	18 (42.9)	0.14
Current nicotine abuse	20 (27.8)	12 (28.6)	0.93
Sleep apnea	1 (1.4)	0 (0)	1.00
Previous stroke/TIA	10 (13.9)	4 (9.5)	0.57
Dementia	0 (0)	1 (2.4)	0.37
Antidepressant premedication, *n* (%)	3 (4.2)	3 (7.1)	0.67
**Stroke-related data**			
NIHSS, median (IQR)			
Total NIHSS score	8 (6)	5 (3)	0.002
Motor items of NIHSS	4 (5)	2 (2)	0.001
Acute therapy, *n* (%)	39 (54.2)	18 (42.9)	0.24
Intravenous thrombolysis	20 (51.3)	6 (33.3)	
Endovascular therapy	7 (17.9)	7 (38.9)	
Combinatory treatment	12 (30.8)	5 (27.8)	0.21
Stroke etiology, TOAST-classification, *n* (%)			0.53
Large-artery atherosclerosis	24 (33.3)	13 (31.0)	
Cardio embolism	24 (33.3)	13 (31.0)	
Small-vessel occlusion	7 (9.7)	5 (11.9)	
Stroke of other determined etiology	0 (0)	2 (4.8)	
Stroke of undetermined etiology	17 (23.6)	9 (21.4)	
ESUS, *n* (%)	15 (20.8)	9 (21.4)	0.94
Interval between admission and study entry, days, median (IQR)	4 (4.25)	3.5 (3.75)	0.039
**Questionnaires**			
Depression, BDI			
Median (IQR)	9 (9)	6.5 (10)	0.052
Any depression (BDI ≥10), *n* (%)	32 (44.4)	15 (35.7)	0.36
Mild depression (10 ≤ BDI ≤ 19)	23 (31.9)	11 (26.2)	0.69
Moderate depression (20 ≤ BDI ≤ 29)	9 (12.5)	4 (9.5)	0.77
MMSE, median (IQR)	24 (9)	25 (8)	0.33

*mRS, modified Rankin Scale; TIA, transient ischaemic attack; NIHSS, National Institutes of Health Stroke Scale; TOAST, trial of ORG 10172 in acute stroke treatment; ESUS, embolic stroke of undetermined source; BDI, Beck Depression Inventory; MMSE, Mini Mental State Exam*.

Median duration of sertraline intake was 82 days (IQR 64–90). Of 72 patients in the sertraline group, 46 patients (63.9%) continued sertraline intake until the telephone interview, whereas 25 patients (34.7%) stopped taking sertraline prematurely. One patient (1.4%) could not provide any information on medication. Most frequent reasons for premature termination were adverse effects (*n* = 8) including changes in personality (*n* = 3), seizure (*n* = 1), hyponatremia (*n* = 1), dizziness (*n* = 1), and cognitive disturbances (*n* = 1) as well as medication change by the treating general practitioner (*n* = 5), and unspecified reasons (*n* = 8).

**Figure 2:**
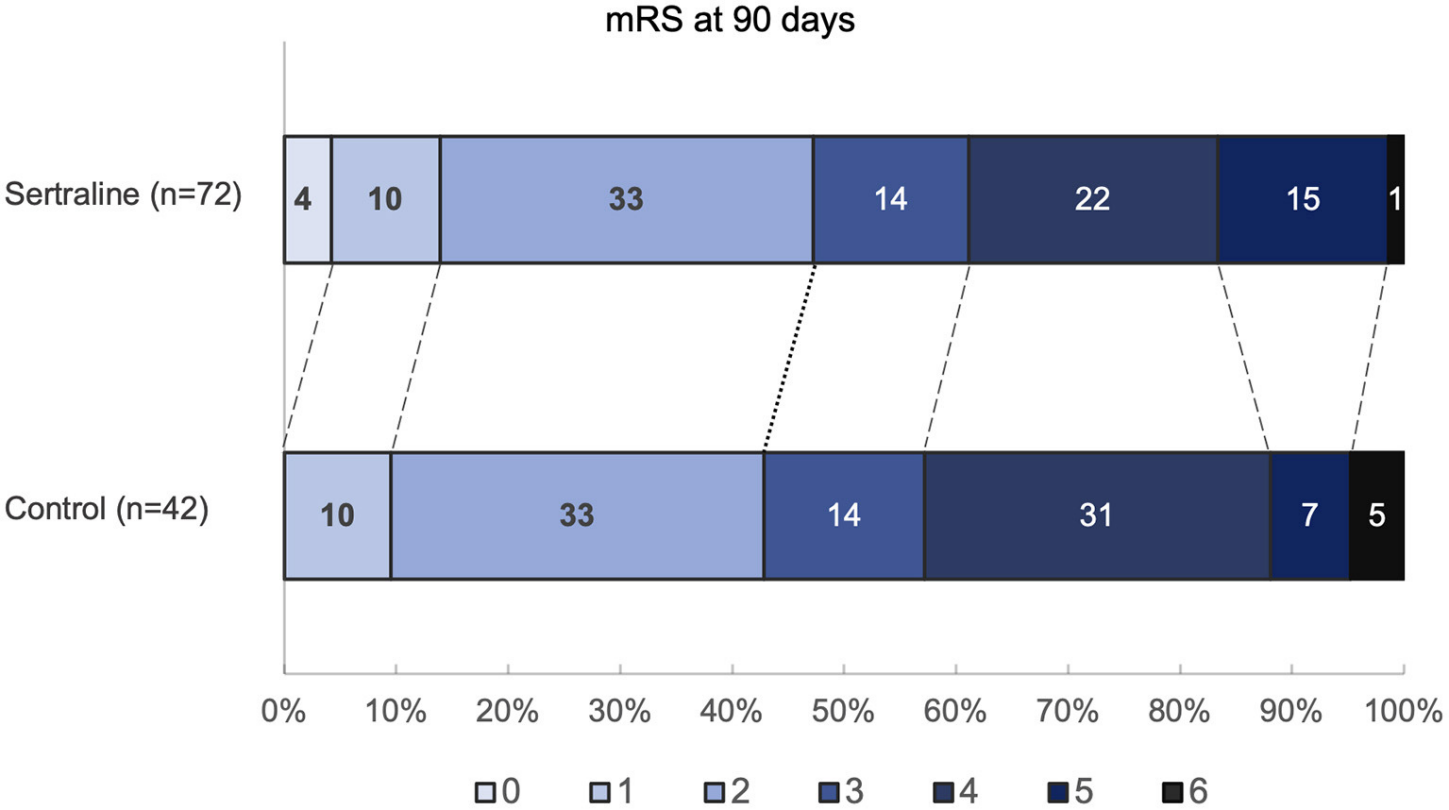
Distribution of mRS scores at 90 days. Values within each colored region correspond to the percentage of patients with the corresponding mRS outcome. mRS indicates modified Rankin Scale.

The mRS scores at 3 months were available in all patients. At 3 months, median mRS scores were comparable between both groups (3 [IQR 2–4] vs. 3 [IQR 2–4]; *p* = 0.67) (Figure 2). Likewise, the frequency of favorable functional outcome did not differ between patients treated with sertraline and controls (34/72 [47.2%] vs. 18/42 [42.9%]; *p* = 0.65). However, a higher proportion of patients improved to a favorable functional status (mRS score of ≤2) between discharge and follow-up in the sertraline group as compared with controls (19/72 [26.4%] vs. 5/42 [11.9%] patients; *p* < 0.001). After adjusting for age and baseline NIHSS, multivariable regression analysis revealed an association between sertraline intake and favorable functional outcome at 3 months (OR 3.10, 95% CI 1.02–9.41, *p* = 0.045). The full model is shown in Table 2. When we added PSD (defined by a BDI ≥10) at follow-up to the model, its presence led to a 65% lower chance of achieving a favorable functional outcome compared to patients without PSD (OR 0.35, 95% CI 0.13–0.97, *p* = 0.043). In this *post-hoc* model, sertraline still showed a trend toward improved favorable functional outcome as compared with controls (OR 3.06, 95% CI 0.99–9.49, *p* = 0.052).

**Table 2:** Multivariable regression analyses of favorable functional outcome and post-stroke depression at 3 months.

**Variable**	**Comparison**	**OR (95% CI)**	***p***
Sertraline	yes vs. no	3.10 (1.02–9.41)	0.045
Age	per 1-year increase	0.88 (0.84–0.93)	<0.001
NIHSS at baseline	per 1-point increase	0.74 (0.63–0.86)	<0.001

*Favorable functional outcome defined as mRS ≤2 constitutes the dependent variable. Variables entered into the model comprised age, baseline NIHSS at study entry, group assignment, and acute therapy. The variable acute therapy was removed from the final model at p = 0.596 (p > 0.2). mRS indicates modified Rankin Scale; NIHSS, National Institutes of Health Stroke Scale; OR, odds ratio; CI, confidence interval*.

There were no differences between the two groups with regard to median PHQ-9 score (5 [IQR 3–8] vs. 6 [IQR 3.5–10], *p* = 0.399) or median BDI score (9 [IQR 5–13] vs. 10 [IQR 5.5–15], *p* = 0.57) at 3 months. Also, there was no difference between the presence of depression (BDI ≥10) after 3 months (26/53 [49.1%] vs. 14/28 [50.0%] patients, *p* = 0.643). Neither there was a difference when PHQ-9 cutoffs were considered (*p* > 0.05). In multivariable regression analysis adjusted for group assignment, age and baseline NIHSS, there was no association between sertraline intake and the presence of PSD at 3 months, neither with PHQ-9 (OR 1.35, 95% CI 0.28–6.4, *p* = 0.708) nor with BDI (OR 0.99, 95% CI 0.37–2.61, *p* = 0.980). Taking into account incident depression at 3 months, however, none of the patients assigned to the sertraline group developed a PSD while five patients in the control group were diagnosed with incident PSD (according to BDI), which corresponds to an increase of 17.9% (*p* = 0.004). Further secondary and safety outcomes are detailed in Table 3.

**Table 3:** Secondary patient outcomes at 3 months.

	**Sertraline**	**Control**	***p***
	**(*n* = 72)**	**(*n* = 42)**	
**Safety outcomes**			
TIA, *n* (%)	0 (0)	0 (0)	1.00
Stroke, *n* (%)	3 (4.2)	0 (0)	0.30
Acute coronary events, *n* (%)	0 (0)	0 (0)	1.00
Death	1 (1.4)	2 (4.8)	0.55
**Questionnaires**			
Return of questionnaires, *n* (%)			
No return	18 (25.0)	14 (33.3)	
Return	54 (75.0)	28 (66.7)	0.28
Complete	46 (83.6)	23 (82.1)	
Elapsed time between phone interview and completed questionnaires, days	*n* = 54	*n* = 26	
Median (IQR)	11 (24)	19.5 (62)	0.22
PHQ-9	*n* = 54	*n* = 28	
Median (IQR)	5 (5)	6 (6.5)	0.40
Presence of depression (PHQ-9 ≥ 10), *n* (%)	10 (18.5)	9 (32.1)	0.18
BDI	*n* = 53	*n* = 28	
Median (IQR)	9 (8)	10 (9.5)	0.57
Presence of any depression (BDI ≥10), *n* (%)	26 (49.1)	14 (50.0)	1.00
Incident depression (BDI ≥10), *n* (%)	0 (0)	5 (17.9)	0.004

*TIA, transient ischemic attack; PHQ-9, Patient Health Questionnaire; BDI, Beck Depression Inventory*.

## Discussion

The major result of this non-interventional study was that early intake of sertraline might favor functional recovery as measured by the mRS in patients with acute ischemic stroke. Moreover, less patients receiving sertraline developed incident depression at 3 months suggesting a potential role for this agent in severely affected stroke patients who are at high risk for PSD.

As opposed to the recently published FOCUS, AFFINITY, and EFFECTS trials, patient eligibility criteria in the present study resembled those applied in the FLAME trial that solely enrolled patients with ischemic stroke (5, 15–17). In pre-clinical studies, potential neuroprotective and neuroplastic effects of SSRI on post-stroke recovery were largely attributed to modification of pathogenic mechanisms following ischemic stroke, such as ischemia-associated hyperexcitation, inflammatory processes in the post-acute phase, augmentation of cerebral blood flow, as well as enhancement of BDNF-expression and stimulation of adult neurogenesis in the subependymal zone and in the hippocampal dentate gyrus (4–10, 12–14). Thus, the predominant inclusion of this stroke subtype may ensure high internal validity in studies on neuroprotective effects of SSRI and stroke. The recent fluoxetine trials, in turn, additionally included patients with intracerebral hemorrhage (ranging from 12 to 15%), who, aside from the aforementioned pathophysiologic considerations, naturally have worse functional prognosis than ischemic stroke patients (15–17, 24).

Although we did not find an absolute difference in the unadjusted analysis of favorable functional outcome at 3 months between both study groups, one should notice that a high proportion of control patients were already functionally independent at discharge leaving less potential for improvement in this group. On the other hand, more than one-fourth of sertraline patients showed substantial improvement in mRS over the first weeks following discharge despite more severe baseline deficits as reflected by both overall and motor NIHSS scores, while no such effects were seen in the control group. Considering these differences in baseline stroke severity, sertraline was associated with a more than 3-fold probability of achieving a favorable functional outcome after 3 months, which is in line with results from a *post-hoc* analysis of the FLAME trial (mRS 0–2 at 3 months: fluoxetine, 26% vs. control, 9%) (5). Although hypothesis generating, sertraline might therefore have a particular effect on motor deficits as previously suggested for fluoxetine by the FLAME but rejected by the FOCUS, AFFINITY, and EFFECTS trials (5, 15–17). However, the median NIHSS scores in the FOCUS and AFFINITY (6 points) as well as in the EFFECTS (3 points) trials indicate a predominance of mild strokes at inclusion, especially in comparison with patients studied in the FLAME trial (12.8 points in the fluoxetine group) (5, 15–17). While patients in our study and in the FLAME trial were functionally independent at baseline, up to 8% of patients in the fluoxetine RCTs trials suffered from functional dependence prior to the index stroke (5, 15–17). The results of these trials might therefore be limited by an insufficient delimitation of patients potentially susceptible to a beneficial effect of SSRI.

According to mechanistic considerations derived from animal experiments, sertraline intake should started as early as possible after the neurologic index event to mediate a neuroprotective effect on cerebral hemodynamics and impede evolution of penumbra into core (8–10, 25, 26). Thus, sertraline was started in our study at a median of 4 days after stroke onset (with almost one-third of patients treated within 3 days from stroke onset) that is far below the corresponding interval in the FLAME trial (mean 8.9 days) (5). The FOCUS, AFFINITY, and EFFECTS trial even defined a much wider treatment interval with a maximum of 15 days after stroke onset (15–17). However, early initiation of SSRI treatment is challenging in the acute phase of stroke. There is general notion that SSRI should not be used routinely to promote recovery after stroke, and depression currently appears the only indication that justifies early treatment with SSRI in stroke patients (18, 22). In the acute phase of stroke, however, depressive symptoms might by obscured by neurological deficits complicating its recognition and the focus is rather on stroke treatment and rehabilitation than evaluation of PSD. The necessity of a fasting phase in acutely treated stroke patients and dysphagia after stroke may also delay initiation of SSRI treatment in acute stroke (27). As potential neuroprotective effects of SSRI appear to be frontloaded in ischemic stroke, future trials need to ensure its early initiation in the hyperacute phase of stroke when vulnerability to irreversible injury is the highest.

One-third of patients discontinued sertraline intake prior to 90-days follow-up, whereas most reasons for premature termination were not necessarily due to pharmacological undesirable effects (except epileptic seizure and hyponatremia in each case). According to available literature data, bone fractures, epileptic seizures and hyponatremia may occur in up to 4% of patients treated with fluoxetine (15–17). Respective data for sertraline in stroke patients are lacking, although similar risks can be assumed for its utilization.

A large fraction (>40%) of patients in this study showed any depressive symptoms at study inclusion (according to a BDI ≥10 points), which might have distorted our negative results regarding a potential preventive effect of sertraline on PSD. This contrasts with numerous studies from the literature that showed preventive effects of certain SSRI on both prevention and treatment of PSD (21, 28, 29). However, when we considered incident depression as the variable of interest, fewer patients taking sertraline were found having incident PSD at 3 months compared with controls, which was seen for fluoxetine in a similar manner in the recent FOCUS and EFFECTS trials (15, 17).

Our study has several limitations. First, as group allocation was not random, our findings might be influenced by selection bias and further potential shortcomings associated with the non-randomized design. Although consecutive sampling was applied in our study, the eventually low number of control patients might be a particular indicator of sampling bias in our study. On the other hand, selection bias might have rather deviated the results toward false negativity as severely affected stroke patients were more likely to be treated with sertraline in our study than less affected patients (as reflected by different NIHSS scores at study entry). A beneficial effect of sertraline, if any, therefore needed to be strong to become apparent in our study. Nonetheless, a potential placebo effect in those treated with sertraline should be considered when interpreting our study results. Second, the high proportion of patients suffering from any degree of depression at study entry complicates the differentiation between potentially neuroprotective effects of sertraline on stroke recovery and its natural antidepressant effect that might have enhanced patients' motivation and cooperation during rehabilitation. Any depressive symptoms at study entry might have increased the odds of having PSD and unfavorable functional outcome at 3 months (22). Nonetheless, the observation that any depressive symptoms were similarly distributed among both groups may have minimized this source of bias in our study. When we adjusted for PSD, sertraline was no longer significantly associated with favorable functional outcome suggesting that adverse effects of PSD might have outweighed potentially beneficial effects of sertraline on functional outcome post-stroke. Consequently, future trials should focus on stroke patients absent of any depressivity at study baseline to explore the sole impact of early sertraline on stroke-recovery. Third, we did not apply psychiatric interviews for depression diagnosis, and the use of self-assessment instruments instead could have deviated the true frequency of PSD in our study, especially when completion of the survey was supported by relatives or others (30). Lastly, our data only allow conclusions to be drawn on the SSRI sertraline. Except fluoxetine that remains the only SSRI tested for clinical efficacy in a large randomized trial, other SSRI such as citalopram showed also promising results on motor outcome in stroke patients and could therefore represent an option for future clinical trials evaluating neuroprotective or neurorestorative therapies (31). The strengths of our study comprise the prospective approach, the well-characterized study population with stroke-associated motor deficits and the completeness of follow-up data concerning the primary endpoint.

## Conclusions

Early intake of sertraline was associated with a tendency toward improved functional recovery from acute ischemic stroke and prevention of incident PSD in these patients. Although limited by its observational nature, our data might form the basis for a confirmatory phase II randomized control trial.

## Data Availability Statement

The raw data supporting the conclusions of this article will be made available by the authors, without undue reservation.

## Ethics Statement

This study was approved by the Ethics Committee (EK) of the Technische Universitaet Dresden (EK 501122016). The patients/participants provided their written informed consent to participate in this study.

## Author Contributions

TS and KB: conceptualization and supervision. KB: methodology and resources. IS and KB: statistical analysis. IS and CH: data curation. IS: writing—original draft preparation. IS, TS, L-PP, CH, JB, HR, VP, and KB: writing—review and editing. All authors have read and agreed to the published version of the manuscript.

## Conflict of Interest

The authors declare that the research was conducted in the absence of any commercial or financial relationships that could be construed as a potential conflict of interest.

## Publisher's Note

All claims expressed in this article are solely those of the authors and do not necessarily represent those of their affiliated organizations, or those of the publisher, the editors and the reviewers. Any product that may be evaluated in this article, or claim that may be made by its manufacturer, is not guaranteed or endorsed by the publisher.
